# Supplementary material for: Predictive language comprehension in Parkinson’s disease
Source: PLoS One. 2023 Feb 8;18(2):e0262504. doi: 10.1371/journal.pone.0262504 (PMC9907838; doi:10.1371/journal.pone.0262504)
Supplement: S1 Table — (PDF) [file pone.0262504.s001.pdf]

S1 Table. Norming Studies Participant Demographics.

Variable	*M* (SD)<br>95% Confidence Interval [Lower, Upper]<br>Range		
Study 1	***Younger-old***	***Older-old***	**t-tests**
**Age (yrs.)**	**60.64** (6.31)<br>[57.00, 64.28]<br>52 - 68	**75.46** (3.84)<br>[73.14, 77.78]<br>70 - 80	$t(25) = -7.30$,<br>$p < .001$
**Education (yrs.)**	**17.64** (3.27)<br>[15.75, 19.53]<br>14 - 27	**17.58** (2.10)<br>[16.25, 18.92]<br>13 – 21.5	$t(25) = 0.05$,<br>$p = .957$
**MoCA (/30)**	**28.14** (1.41)<br>[27.33, 28.95]<br>25 - 30	**26.62** (1.98)<br>[25.42, 27.81]<br>23 - 29	$t(25) = 2.32$,<br>$p = .029$
Study 2			
**Age (yrs.)**	**58.41** (5.96)<br>[55.34, 61.48]<br>50 - 67	**78.38** (5.35)<br>[75.15, 81.62]<br>70 - 87	$t(28) = -9.50$,<br>$p < .001$
**Education (yrs.)**	**17.94** (2.68)<br>[16.56, 19.32]<br>15 - 27	**18.38** (2.27)<br>[17.02, 19.75]<br>14.5 - 23	$t(28) = -0.48$,<br>$p = .635$
**MoCA (/100%)**	**93.94** (4.27)<br>[91.66, 96.21]<br>86.4 - 100	**90.70** (5.21)<br>[87.55, 90.70]<br>81.8 – 96.7	$t(28) = 2.32$,<br>$p = .077$

Note: In study 1, participants' MoCA scores ranged from 23-30, M = 27.41, SD = 1.85.
In study 2, participants' MoCA percentage scores ranged from 81.8-100%, M = 92.49, SD = 4.91. In the telephone version of the MoCA (norming study 2), a score of 22 = a score of 30 on the in-person version of the MoCA, and the suggested cutoff score for normal cognition is 18 (81.8% correct).
