# Supplementary material for: Predictive language comprehension in Parkinson’s disease
Source: PLoS One. 2023 Feb 8;18(2):e0262504. doi: 10.1371/journal.pone.0262504 (PMC9907838; doi:10.1371/journal.pone.0262504)
Supplement: S2 Table — (PDF) [file pone.0262504.s002.pdf]

S2 Table. Neuropsychological Battery.

Domain	*Task Name*	*Task Description*	*Cognitive Skills*
Executive Function and Attention	D-KEFS Trail Making Test[1]	Participants draw a line from number to letter in ascending order, alternating between numbers and letters	Visual-motor sequencing task; task-switching
	D-KEFS Color Word Interference Test	Words written in different colour than word written. Participants switch between reading written word and saying font colour.	Ability to inhibit a dominant and automatic verbal response
	CLOX	Participants construct an analogical clock first from memory (CLOX 1), then again by copying the experimenter's clock (CLOX 2).	Visuospatial processing and planning
	Digit Span	Participants recite digit spans first forwards then backwards.	Short-term and working memory
	Semantic Fluency (Category—animals)	Participants list as many animals as they can within 60 seconds	Lexical access; updating ability
Language	Boston Naming Test (short form)[1]	Noun confrontation naming	Lexical retrieval of imageable nouns (high and low frequency of occurrence)
	Northwestern Naming Battery	Verb confrontation naming & Verb comprehension	Production and identification of imageable verbs
	Northwestern Assessment of Verbs and Sentences	Participants select images that match spoken sentences of varying syntactic complexity	Sentence comprehension
	Pyramids and Palm Trees (short form)[2]	Participants identify which of two objects "goes with" a target	Thematic relatedness
Memory	Hopkins Verbal Learning Test	Auditory learning of a list of 12 words.	Verbal learning and memory
	Brief Visuospatial Memory Test-Revised (BVMT-R)	Visual learning of 6 different designs	Visuospatial learning and memory

*Note: Reference List

[1]Graves, Bezeau, Fogarty, & Blair, 2004; [2]Breining…Hillis, 2015

S3 Appendix. Visual Stimuli Selection and Editing.
