# Supplementary material for: Predictive language comprehension in Parkinson’s disease
Source: PLoS One. 2023 Feb 8;18(2):e0262504. doi: 10.1371/journal.pone.0262504 (PMC9907838; doi:10.1371/journal.pone.0262504)
Supplement: S3 Table — (PDF) [file pone.0262504.s003.pdf]

S5 Table. Predictive Sentences Practice.

Sentence	Target	Distractor 1	Distractor 2	Distractor 3
The maid spreads the blanket.	blanket	broom	icing	cake
The hunter loads the rifle.	rifle	antlers	groceries	register
The gambler throws the dice.	dice	jackpot	soccer ball	goal
The soldier writes the letter.	letter	cannon	experiment	rocket
The photographer cleans the camera.	camera	photo	newspaper	typewriter
The doctor reads the x-ray.	x-ray	injection	story	assignment
The butcher wraps the steak.	steak	scale	candy cane	sleigh
The ringmaster trains the lion.	lion	cage	runner	stopwatch
