# Supplementary material for: Predictive language comprehension in Parkinson’s disease
Source: PLoS One. 2023 Feb 8;18(2):e0262504. doi: 10.1371/journal.pone.0262504 (PMC9907838; doi:10.1371/journal.pone.0262504)
Supplement: S4 Table — (PDF) [file pone.0262504.s004.pdf]

S6 Table. Predictive Sentences, Full Trials (Sets 1-12).

Audio Sentence	Target Image	Agent-related distractor	Action-related distractor	Unrelated distractor
The fisherman sews the net.	net	boat	quilt	cradle
The fisherman rocks the boat.	boat	net	cradle	quilt
The grandmother rocks the cradle.	cradle	quilt	boat	net
The grandmother sews the quilt.	quilt	cradle	net	boat
The boxer fastens the headgear.	headgear	punching bag	seatbelt	brakes
The boxer hits the punching bag. (H)	punching bag	headgear	brakes	seatbelt
The driver fastens the seatbelt.	seatbelt	brakes	headgear	punching bag
The driver hits the brakes. (H)	brakes	seatbelt	punching bag	headgear
The baby drinks the milk.	milk	bib	champagne	heels
The baby wears the bib.	bib	milk	heels	champagne
The bride drinks the champagne.	champagne	heels	milk	bib
The bride wears the heels.	heels	champagne	bib	milk
The baker rolls the dough.	dough	oven	sleeping bag	lantern
The baker lights the oven.	oven	dough	lantern	sleeping bag
The camper rolls the sleeping bag.	sleeping bag	lantern	dough	oven
The camper lights the lantern.	lantern	sleeping bag	oven	dough
The child catches the football. (H)	football	lollipop	mouse	paw
The child licks the lollipop.	lollipop	football	paw	mouse
The cat catches the mouse. (H)	mouse	paw	football	lollipop

The cat licks the paw.	paw	mouse	lollipop	football
The horse eats the carrot.	carrot	fence	fish	waves
The horse jumps the fence. (H)	fence	carrot	waves	fish
The dolphin eats the fish.	fish	waves	carrot	fence
The dolphin jumps the waves. (H)	waves	fish	fence	carrot
The policeman closes the cell.	cell	motorcycle	stable	horse
The policeman rides the motorcycle.	motorcycle	cell	horse	stable
The jockey closes the stable.	stable	horse	cell	motorcycle
The jockey rides the racehorse.	horse	stable	motorcycle	cell
The lifeguard scans the beach.	beach	swimmer	aisle	money
The lifeguard saves the swimmer. (H)	swimmer	beach	money	aisle
The shopper scans the aisle.	aisle	money	beach	swimmer
The shopper saves the money.	money	aisle	swimmer	beach
The detective crosses the courtroom. (H)	courtroom	fugitive	jungle	zebra
The detective hunts the fugitive. (H)	fugitive	courtroom	zebra	jungle
The lion crosses the jungle. (H)	jungle	zebra	courtroom	fugitive
The lion hunts the zebra. (H)	zebra	jungle	fugitive	courtroom
The mechanic pours the oil.	oil	tools	salt	dishes
The mechanic organizes the tools. (H)	tools	oil	dishes	salt
The waitress organizes the dishes. (H)	dishes	salt	tools	oil
The waitress pours the salt.	salt	dishes	oil	tools

The pilot wears the uniform.	uniform	airplane	overalls	tractor
The pilot controls the airplane.	airplane	uniform	tractor	overalls
The farmer wears the overalls.	overalls	tractor	uniform	airplane
The farmer controls the tractor.	tractor	overalls	airplane	uniform
The pirate wears the eyepatch.	eyepatch	ship	sunglasses	car
The pirate steers the ship.	ship	eyepatch	car	sunglasses
The chauffeur wears the sunglasses.	sunglasses	car	eyepatch	ship
The chauffer steers the car.	car	sunglasses	ship	eyepatch
