# Supplementary material for: Predictive language comprehension in Parkinson’s disease
Source: PLoS One. 2023 Feb 8;18(2):e0262504. doi: 10.1371/journal.pone.0262504 (PMC9907838; doi:10.1371/journal.pone.0262504)
Supplement: S5 Table — (PDF) [file pone.0262504.s005.pdf]

S7 Table. Baseline Sentence Practice.

Target	Distractor 1	Distractor 2	Distractor 3
diamond	hat	flower	beer
guitar	deer	ladder	brick
jacket	bow	dog	pine cone
banana	pan	umbrella	lightbulb
bike	present	mushroom	fork
