# Supplementary material for: Predictive language comprehension in Parkinson’s disease
Source: PLoS One. 2023 Feb 8;18(2):e0262504. doi: 10.1371/journal.pone.0262504 (PMC9907838; doi:10.1371/journal.pone.0262504)
Supplement: S6 Table — (PDF) [file pone.0262504.s006.pdf]

S8 Table. Baseline Trials.

Target	Distractor 1	Distractor 2	Distractor 3
helmet	clock	balloon	log
drum	bathtub	rope	strawberry
tape	strawberry	drum	helmet
teapot	fan	brush	drum
bathtub	violin	dress	slide
lamp	feather	strawberry	truck
feather	belt	slide	teapot
log	scarf	fan	tape
scarf	violin	teapot	roller skate
slide	log	feather	balloon
dress	flashlight	clock	mailbox
clock	fan	tape	shovel
brush	flashlight	lamp	pumpkin
pumpkin	balloon	rope	violin
rope	teapot	scarf	flashlight
shovel	balloon	pumpkin	lamp
mailbox	strawberry	truck	belt
roller skate	flashlight	squirrel	brush
truck	violin	shovel	dress
squirrel	roller skate	belt	fan
