# Supplementary material for: Predictive language comprehension in Parkinson’s disease
Source: PLoS One. 2023 Feb 8;18(2):e0262504. doi: 10.1371/journal.pone.0262504 (PMC9907838; doi:10.1371/journal.pone.0262504)
Supplement: S7 Table — (PDF) [file pone.0262504.s007.pdf]

**S9 Table. Analyses of PD versus control gaze logits to the target object during predictive sentences.**

| | Agent Time Window | | | Verb Window | | | Target Window | | |
|---|---|---|---|---|---|---|---|---|---|
| | *Estimate* | *S.E.* | *p* value | *Estimate* | *S.E.* | *p* value | *Estimate* | *S.E.* | *p* value |
| Intercept | -1.441 | 0.10 | **< .001** | -0.353 | 0.11 | **< .01** | 0.662 | 0.18 | **< .001** |
| Linear time | 2.342 | 0.20 | **< .001** | 0.545 | 0.12 | **< .001** | 0.998 | 0.24 | **< .001** |
| Quadratic time | -0.659 | 0.11 | **< .001** | 0.061 | 0.05 | 0.270 | -0.295 | 0.08 | **< .001** |
| Group (Control/PD) | 0.086 | 0.13 | 0.505 | 0.128 | 0.15 | 0.386 | 0.263 | 0.32 | 0.409 |
| Group x Linear | -0.216 | 0.28 | 0.440 | -0.061 | 0.18 | 0.730 | 0.318 | 0.35 | 0.360 |
| Group x Quadratic | -0.020 | 0.17 | 0.903 | -0.112 | 0.11 | 0.305 | -0.078 | 0.13 | 0.555 |

Note: Bolded values are significant at the $p$ <.05 level
