# Supplementary material for: Predictive language comprehension in Parkinson’s disease
Source: PLoS One. 2023 Feb 8;18(2):e0262504. doi: 10.1371/journal.pone.0262504 (PMC9907838; doi:10.1371/journal.pone.0262504)
Supplement: S8 Table — (PDF) [file pone.0262504.s008.pdf]

S10 Table. Analyses of PD versus control gaze logits to the agent-related object during the predictive window.

	Verb Time Window		
	Estimate	*S.E.*	*p* value
Intercept	-0.902	0.11	**< .001**
Linear time	-0.572	0.13	**< .001**
Quadratic time	-0.252	0.06	**< .001**
Group (Control/PD)	-0.049	0.12	0.690
Group x Linear	-0.120	0.21	0.570
Group x Quadratic	-0.162	0.12	0.166

Note: Bolded values are significant at the p <.05 level
