# Supplementary material for: Predictive language comprehension in Parkinson’s disease
Source: PLoS One. 2023 Feb 8;18(2):e0262504. doi: 10.1371/journal.pone.0262504 (PMC9907838; doi:10.1371/journal.pone.0262504)
Supplement: S9 Table — (PDF) [file pone.0262504.s009.pdf]

S11 Table. Analyses of PD versus control gaze logits to the verb-related object during the combined verb + target window.

	Combined End Time Window		
	Estimate	*S.E.*	*p* value
Intercept	-2.266	0.12	**< .001**
Linear time	-0.616	0.35	0.079
Quadratic time	-1.099	0.27	**< .001**
Group (Control/PD)	-0.042	0.20	0.836
Group x Linear	0.114	0.55	0.837
Group x Quadratic	-0.547	0.34	0.108

Note: Bolded values are significant at the p <.05 level
