# Supplementary material for: Predictive language comprehension in Parkinson’s disease
Source: PLoS One. 2023 Feb 8;18(2):e0262504. doi: 10.1371/journal.pone.0262504 (PMC9907838; doi:10.1371/journal.pone.0262504)
Supplement: S10 Table — (PDF) [file pone.0262504.s010.pdf]

S12 Table. Effect of motion content on gaze logits to the target entity during predictive sentences.

Fixed Effects	Predictive Sentences: Fixations on the Target Object								
	Agent Time Window			Verb Window			Target Window		
	Estimate	*S.E.*	*p* value	*Estimate*	*S.E.*	*p* value	*Estimate*	*S.E.*	*p* value
Intercept	-1.486	0.11	**< .001**	-0.351	0.12	**<.01**	0.624	0.18	**<.001**
Linear time	2.356	0.22	**< .001**	0.457	0.13	**<.001**	0.953	0.26	**<.001**
Quadratic time	-0.609	0.13	**< .001**	0.011	0.06	0.865	-0.342	0.09	**<.001**
Group (Control/PD Intercept)	-0.003	0.13	0.981	0.161	0.15	0.277	0.277	0.32	0.388
Motion Content (Intercept)	-0.188	0.18	0.284	-0.005	0.20	0.982	-0.157	0.18	0.368
Group x Linear	-0.194	0.30	0.510	0.057	0.19	0.758	0.506	0.35	0.152
Group x Quadratic	-0.073	0.19	0.703	-0.211	0.13	0.098	-0.030	0.15	0.842
Motion x Linear	0.042	0.36	0.908	-0.338	0.22	0.129	-0.199	0.40	0.615
Motion x Quadratic	0.181	0.23	0.427	-0.182	0.13	0.152	-0.188	0.17	0.272
Group x Motion (Intercept)	-0.336	0.09	**<.001**	0.091	0.08	0.261	0.034	0.07	0.646
Group x Motion x Linear	0.038	0.35	0.913	0.435	0.26	0.089	0.736	0.28	**<.01**
Group x Motion x Quadratic	-0.271	0.33	0.407	-0.411	0.25	0.106	0.237	0.27	0.386

Note: Bolded values are significant at the p <.05 level
