# Supplementary figures and images for: Predictive language comprehension in Parkinson’s disease
Source: PLoS One. 2023 Feb 8;18(2):e0262504. doi: 10.1371/journal.pone.0262504 (PMC9907838; doi:10.1371/journal.pone.0262504)

S13 Figure. Looks to target versus distractor images in predictive sentences, in logits.

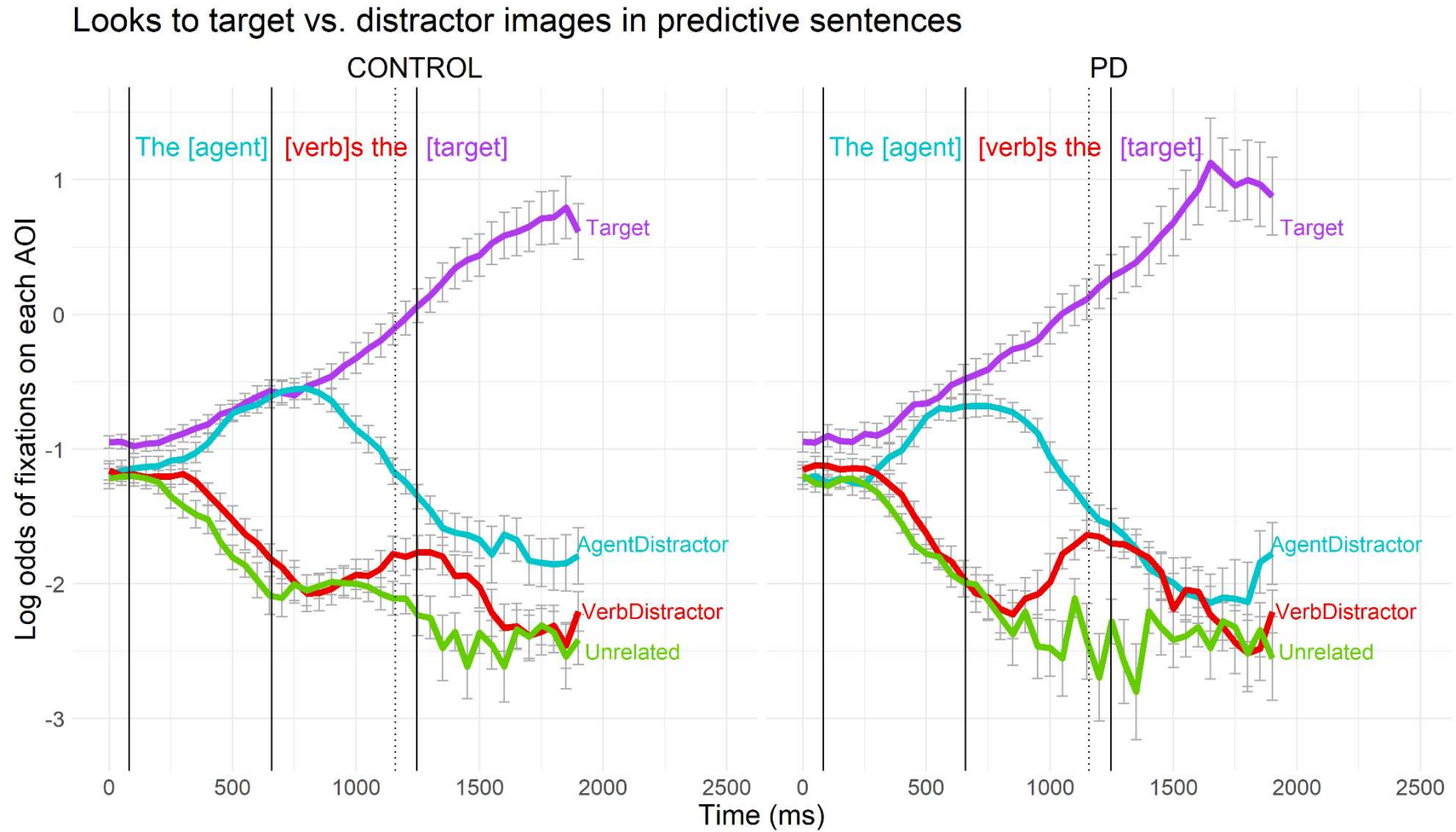

Supplement: S1 Fig — (PDF) [file pone.0262504.s011.pdf]

S14 Figure. Looks to target versus distractor images in baseline sentences, in logits.

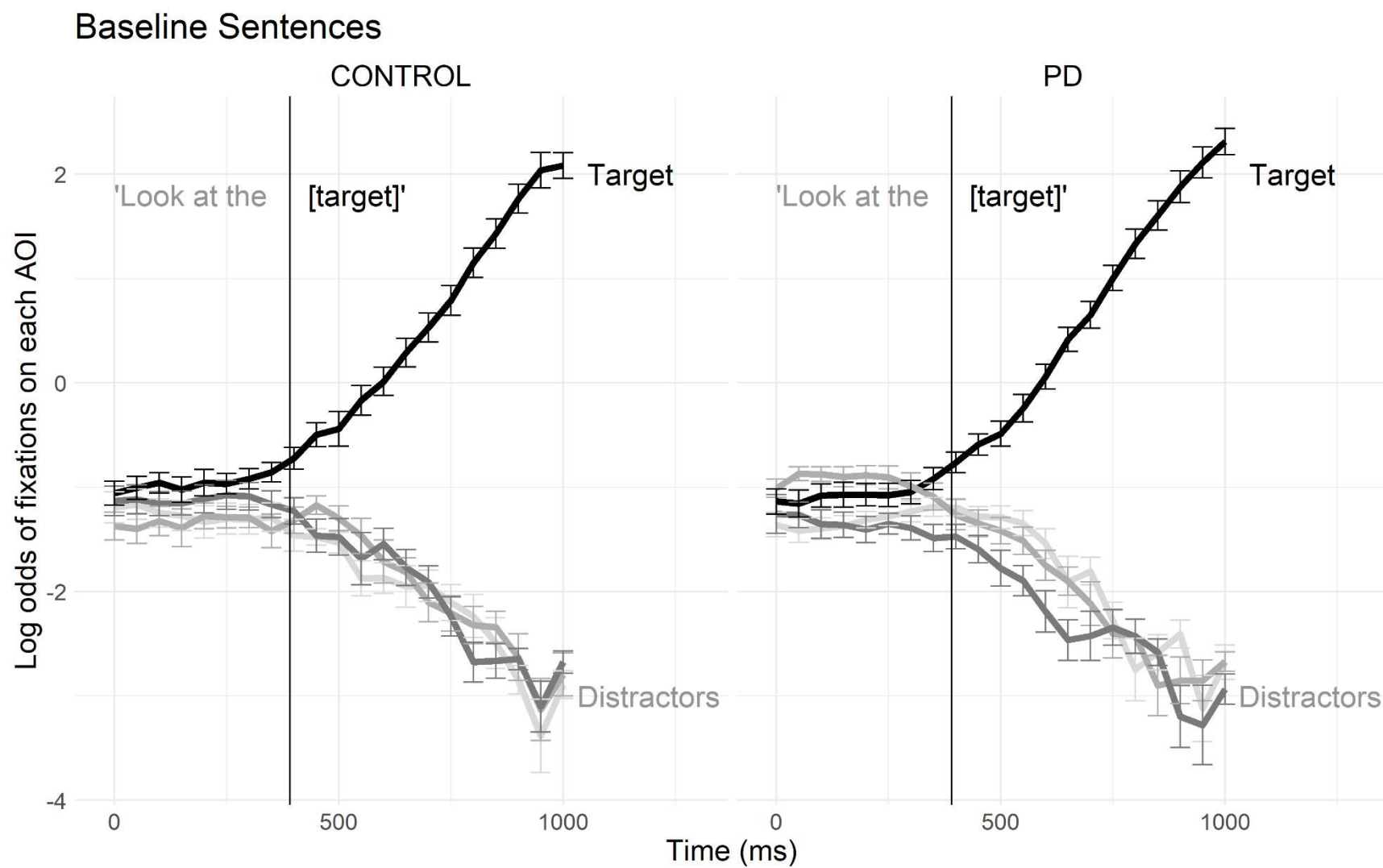

Supplement: S2 Fig — (PDF) [file pone.0262504.s012.pdf]
