# Supplementary material for: Predictive language comprehension in Parkinson’s disease
Source: PLoS One. 2023 Feb 8;18(2):e0262504. doi: 10.1371/journal.pone.0262504 (PMC9907838; doi:10.1371/journal.pone.0262504)
Supplement: S1 Appendix — (PDF) [file pone.0262504.s013.pdf]

S3 Appendix. Visual Stimuli Selection and Editing.

Visual stimuli were edited with Adobe Photoshop to normalize the images. For example, words, brand logos, and vibrant patterns were removed; harsh colors including reds or bright blues were toned down in saturation; and where possible, images were placed onto a plain white background with all shadows and reflections were removed. Photos were edited to a uniform size of 400 by 400px. Multiple options were compiled for each object, and a team of voters unfamiliar with the study design identified which images they found most representative of each object. When possible, images were sourced from image banks intended for use in cognitive research (Brady, et al., 2008; Brodeur, et al., 2010; Kovalenko, et al., 2012; Moreno-Martínez & Montoro, 2012).
