# Supplementary material for: Predictive language comprehension in Parkinson’s disease
Source: PLoS One. 2023 Feb 8;18(2):e0262504. doi: 10.1371/journal.pone.0262504 (PMC9907838; doi:10.1371/journal.pone.0262504)
Supplement: S2 Appendix — (PDF) [file pone.0262504.s014.pdf]

S4 Appendix. Word Onsets in Sentence Audio.

The speaker recorded each sentence three times using flat but natural prosody. The selection of these recordings was determined through consensus ratings of three listeners (four raters in cases of a three-way tie) who selected the overall best file based on the sentences' naturalness and lack of audio distortions or interfering noises. We used Praat (Boersma & Weenink, 2020) to scale the selected sound files to an average intensity of 70 dB. Word onsets and offsets were demarcated in Praat by the first author. Where coarticulation was present, word onsets were marked conservatively, i.e. at the earliest place the next phoneme/word onset was clearly identifiable. Word durations were then manipulated in Praat with a custom script that made use of the overlap-add function to shorten or extend the durations of individual words (Moulines & Charpentier, 1990). As a result of this manipulation, the onsets of each word in each sentence occurred at a predefined time based on the original mean duration of the agents, verbs, etc. (see below table). For the predictive sentences, we then tested three overall sentence durations and selected the duration that sounded most natural to listeners unfamiliar with the study design. Sound onsets were set to 50ms across sentence types to allow sufficient time for audio buffering. For predictive sentences, the final speaking rate was 2.63 words per second. For the non-predictive sentences, the final speaking rate was 4.14 words per second, as most words in these sentences were monosyllabic.

**Prediction Trials**					
	The	***[agent]***	***[verb]s***	***the***	***[target]***
**Onset (ms)**	50	132	708	1171	1296
**Duration (ms)**	82	576	463	125	654
**Non-Prediction Trials**					
		Look	***at***	***the***	***[target]***
**Onset (ms)**		50	233	365	441
**Duration (ms)**		183	132	76	575
