# Supplementary material for: Predictive language comprehension in Parkinson’s disease
Source: PLoS One. 2023 Feb 8;18(2):e0262504. doi: 10.1371/journal.pone.0262504 (PMC9907838; doi:10.1371/journal.pone.0262504)
Supplement: Attachment — Submitted filename: Response to Reviewers PD lang prediction Dec2021.docx [file pone.0262504.s016.docx]

Daniel Mirman, PhD

Academic Editor
PLOS ONE

Dear Dr. Mirman,

Thank you for inviting us to re-submit a revised draft of our manuscript entitled "Predictive language comprehension in Parkinson’s disease", PONE-D-21-22350, to PLOS ONE. We appreciate the continued support and feedback provided by you and each of the reviewers. Thus, it is with great pleasure that we resubmit our article for further consideration. We have incorporated changes that reflect the detailed suggestions you have graciously provided. We also hope that our edits and the responses we provide below satisfactorily address all the issues and concerns you and the reviewers have noted.

To facilitate your review of our revisions, the following is a point-by-point response to the questions and comments delivered in your letter dated 11/9/2021. Page and line numbers refer to the ‘Manuscript’ version without visible tracked changes.

**EDITOR SUGGESTIONS:**

1. Please review your reference list to ensure that it is complete and correct. If you have cited papers that have been retracted, please include the rationale for doing so in the manuscript text, or remove these references and replace them with relevant current references. Any changes to the reference list should be mentioned in the rebuttal letter that accompanies your revised manuscript. If you need to cite a retracted article, indicate the article’s retracted status in the References list and also include a citation and full reference for the retraction notice.
   - **RESPONSE:** We have adjusted referenced article titles to follow sentence capitalization consistently. We have also corrected the title in reference #44 (Copland et al. 2000).

**REVIEWER 1 COMMENTS:**

2. The system will not let me bypass the minimum character count, so I will add that I appreciate the efforts put into running additional analyses and integrating the comments in the revised version. The paper raises more questions than it answers, but suggestions for future studies are relevant and intriguing.

- **RESPONSE:** We would like to take this opportunity to thank this reviewer again for their comprehensive and insightful comments on the previous draft.

**REVIEWER 2 COMMENTS:**

3. The discussion is now clearer about the limitations of the motion results (e.g., “we interpret these results cautiously”; p39ln819). However, this isn’t reflected in the abstract, where these results receive considerable attention. Rather, I encourage acknowledging these limitations and/or limiting the discussion of these results in the abstract. Adding to this discussion (e.g., “it is unclear why a significant motion content x group interaction occurred before the onset of the verb”; p39ln821), I also encourage being explicit that these results are problematic because they imply that participants were sensitive to the content of the verbs before they’d heard them. (As a very minor comment, I think only one sentence type is described in the abstract before, “in either sentence type”; p2ln45.).
   - **RESPONSE:** We appreciate this reviewer’s concern and have revised the abstract (page 2, lines 52-4) and the discussion of the results (page 39, lines 834-6) accordingly. We have also revised page 2, line 45 to omit the implied reference to another sentence type.
4. The verb-related distractor analyses are difficult to interpret without a baseline comparison. Paralleling the target vs. agent-related distractor t-test (p32ln710), I encourage including an analysis like a verb-related distractor vs. unrelated t-test. I also encourage reporting relevant descriptive statistics (M/SD). (As a very minor comment, I wonder if the Baseline analyses shouldn’t be presented before the Predictive analyses; as is, they almost seem unnecessary.)
   - **RESPONSE:** We have completed an additional t-test comparing looks to the verb-related and unrelated distractors (page 34, lines 735-9) and have updated page 32, lines 717-8 to include the requested descriptive statistics (M/SD). Although we appreciate this reviewer’s comment concerning the placement of the baseline sentences, we believe it best to present the predictive sentences first based on readers’ anticipated interest in the research questions addressed by these analyses and to follow with the baseline sentences in case of lingering questions about the reliability of the obtained eye-tracking results.
5. Finally, I also encourage using graphs to capture the growth curve results in a clearer and more compelling way. For example, graphs depicting the measure under analysis (i.e., “our models predict the odds ratio of fixations on the target versus fixations on all other distractors. This odds ratio is log-transformed into “logits” of fixations on each interest area”; p28ln628) would be informative. Likewise, graphs depicting the growth curve fits would also be informative. Rather, it’s not clear how the intercept, linear, etc. growth curve results (e.g., see Table 2) map onto proportions of fixations (e.g., see Figure 2). (As a minor comment, the sample size was 48 participants, which seems at odds with, “when sample sizes are sufficiently large, e.g. >200 for linear estimates and >1000 for quadratic estimates, as was the case in the present study”; p29ln648.)
   - **RESPONSE:** We thank the reviewer for this suggestion and have created two new figures (Figs 3 and 6 in the current manuscript) that depict model fits versus data in logit units. We have also created two new supplemental files (S13 and S14 Figures) that depict Figures 2 and 5 in logit units, although we have chosen not to replace Figures 2 and 5 in the main body of the manuscript because we feel that many readers will find the proportion scale more familiar and intuitive for graphical interpretation. Per the reviewer’s comment about the apparent mismatch in sample size, we have revised page 29, lines 649-651 to clarify.

Again, thank you for giving us the opportunity to strengthen our manuscript with your valuable comments and queries. We have worked hard to incorporate your feedback and hope that these revisions persuade you to accept our submission.
